# More than expected: extracellular waveforms and functional responses in monkey LGN

**DOI:** 10.1101/2023.11.22.568065

**Published:** 2024-05-16

**Authors:** Shi Hai Sun, Nathaniel J. Killian, John S. Pezaris

**Affiliations:** 1 Department of Neurosurgery, Massachusetts General Hospital, Boston, MA, USA; 2 Department of Neurosurgery, Harvard Medical School, Boston, MA, USA; 3 Department of Neurological Surgery, Albert Einstein College of Medicine, Bronx, NY, USA

## Abstract

Unlike the exhaustive determination of cell types in the retina, key populations in the lateral geniculate nucleus of the thalamus (LGN) may have been missed. Here, we have begun to characterize the full range of extracellular neuronal responses in the LGN of awake monkeys using multi-electrodes during the presentation of colored noise visual stimuli to identify any previously overlooked signals. Extracellular spike waveforms of single units were classified into seven distinct classes, revealing previously unrecognized diversity: four negative-dominant classes that were narrow or broad, one triphasic class, and two positive-dominant classes. Based on their mapped receptive field (RF), these units were further categorized into either magnocellular (*M*), parvocellular (*P*), koniocellular (*K*), or non-RF (*N*). We found correlations between spike shape and mapped RF and response characteristics, with negative and narrow spiking waveform units predominantly associated with *P* and *N* RFs, and positive waveforms mostly linked to *M* RFs. Responses from positive waveforms exhibited shorter latencies, larger RF sizes, and were associated with larger eccentricities in the visual field than the other waveform classes. Additionally, *N* cells, those without an estimated RF, were consistently responsive to the visually presented mapping stimulus at a lower and more sustained rate than units with an RF. These findings suggest that the LGN cell population may be more diverse than previously believed.

## Introduction

The lateral geniculate nucleus (LGN) of the thalamus serves as a pivotal hub for processing visual information in mammals, acting as the primary relay station between the retina and the visual cortex (Sherman & Guillery, 2006; Solomon & Lennie, 2007). In primates, the LGN is anatomically segregated into distinct layers, namely magnocellular (M), parvocellular (P), and koniocellular (K). Electrophysiological studies in primates have delineated the functional characteristics of these layers: M neurons exhibit rapid temporal dynamics, well suited for motion detection; P neurons are sensitive to chromatic dynamics, well suited for color and form processing; and K neurons are responsive to short-wavelength (“blue”) photoreceptor inputs (Wiesel & Hubel, 1966; Schiller & Malpeli, 1978; Kaplan & Shapley, 1982; Derrington & Lennie, 1984; Hubel & Livingstone, 1990; Maunsell et al., 1999; Reid & Shapley, 2002; Tailby et al., 2008).

Despite the extensive characterization of these divisions (with K neurons being comparatively less studied), much of our understanding of LGN function stems from electrophysiological recordings employing single-channel electrodes to discern neuronal responses in the form of extracellular spikes. Typically, these signals are biphasic with a dominantly negative voltage excursion, allowing for tentative classification of neuronal cell types: broader waveforms are indicative of excitatory neurons, while narrower waveforms suggest inhibitory neurons (Henze et al., 2000). However, recent advancements in dense multi-electrode arrays and sophisticated spike-sorting algorithms have unveiled a spectrum of waveform shapes across various species and brain regions, diverging from the traditional biphasic and negative waveforms.

These waveform variations include triphasic-spiking waveforms found in rat hippocampus (Barry, 2015) and superior colliculus (Sibille et al., 2022); positive-spiking and long-broad negative waveforms found in the cat visual cortex (Gold et al., 2009; Sun et al., 2021) and human prefrontal cortex (Paulk et al., 2022); and doublet-spiking waveforms consisting of two short downward deflections found in ferret LGN (Murphy et al., 2020). Such discoveries challenge the conventional understanding of extracellular signals and underscore the limitations of single-electrode sampling biases (Olshausen & Field, 2005; Talebi & Baker, 2016), potentially leading to the oversight of crucial neuronal populations. Identifying the gamut of extracellular signals from the LGN may help in understanding the processing in the early visual pathway.

Thus, in this study, we agnostically survey the extracellular space in the LGN of rhesus macaques through recordings that were not optimized for single-unit isolation, employing a variety of stimulus and electrode properties. For each recorded LGN unit, we characterize its receptive field (RF) and extracellular spike waveform classes, alongside several response metrics, to elucidate potential relationships between spike shape and neuronal identity. Our investigation also unveils a subset of units lacking an estimated RF (termed non-RF, or N units), an occurrence rarely reported in existing literature, with most of these units exhibiting consistent responsiveness to the visual stimulus at a lower and more sustained response rate to units with an RF. The presence of N cells suggests a more nuanced complexity in LGN processing beyond the classical M, P, and K divisions, and hints at undiscovered computational mechanisms within the visual system. Our findings challenge the conventional paradigm of LGN organization and underscore the necessity for a fuller understanding of neural populations within this critical visual relay station. By delineating the LGN’s neural landscape beyond traditional classifications, our study contributes to advancing knowledge of early visual processing mechanisms and the interpretation of extracellular signals.

## Methods

### Ethics

Recordings were made from three awake adult rhesus monkeys (*Macaca mulatta*, 3M, 19–20 kg). The animals were maintained in the AAALAC accredited animal facility at the Massachusetts General Hospital. All research procedures were approved by the Massachusetts General Hospital Institutional Animal Care and Use Committee (IACUC) and were carried out according to the NIH Guide for the Care and Use of Laboratory Animals.

### Animal preparation

The animals were surgically implanted with custom titanium head-holding posts and recording cylinders that allowed bilateral access to the LGN. Animals were trained to sit in a primate chair (B & M Plastics, Inc.), and placed in a shielded recording chamber (Crist Instrument Co.) during recordings. The animals were positioned so that their eyes were 43 cm from a 22-inch CRT monitor (Viewsonic P220f) with neutral gaze position near the center of the monitor.

Gaze location was monitored by an infrared video camera at a 500 Hz sampling rate (High-Speed Primate, SensoMotoric Instruments GmbH), with infrared illuminators stacked to yield a single corneal reflection. Software (iView X, SensoMotoric Instruments) was used to adjust the gains and offsets of the pupil-corneal reflection distance. Gaze position signals were then sent via analog signals to a behavioral control computer, where additional calibration was performed (2D quadratic fit to nine calibration points). See Figure 1A for a schematized depiction of the recording cylinder, monitor positioning and eye tracking.

### Behavioral task

A trial-based task was used to map the response fields of neurons (Figure 1B). To begin each trial, the animal was required to fixate on a centrally presented small, circular target (0.2° diameter white spot) for 400 ms. The visual mapping stimulus then began with the fixation point overlaid on the animated noise so that it continued to be visible, and the animal was required to continue maintaining fixation within a 2.0° diameter circular window for the duration of the stimulus (2.5–5.0 seconds, mode of 2.5 seconds; typical fixation performance was much tighter than this window; see Results). Successful fixations through the entire stimulus were rewarded with drops of sweet liquid and pleasant audio feedback. Data from successful trials were used for mapping analysis (4–215 trials per experiment, median of 39 trials), but data from all trials were used for the spike waveform analysis.

### Visual Stimuli

Custom software (PLECS, Pezaris Lab Experiment Control System) was used to display noise stimuli that spanned both luminance and chrominance spaces, in addition to controlling behavioral state and data collection (Killian et al., 2016). The visual stimuli were presented on a CRT display (Viewsonic P220f) at 160 Hz refresh rate and 8 bits per color channel on a display area of 40 by 30 cm (400 by 300 pixels) that subtended 53° by 40° in visual field. Two classes of noise stimuli were presented: high-resolution 400 by 300 pixel sets with power spectra proportional to one over frequency (five sets, described below) and low-resolution 80 by 60 pixels with a white power spectrum (one set). Each recording used a single mapping stimulus from this collection.

The high-resolution noise stimuli were generated through computations in the frequency domain:

(1) $$\xi =\left(\xi_{x},\xi_{y},\xi_{t}\right)$$

(2) $$S(\xi )=\frac{1}{{∥\xi ∥}^{\beta }}$$

(3) $$\left|(Ff)(\xi ){|}^{2}=S(\xi \right)$$

(4) $$Ff(\xi )=(S(\xi ){)}^{1/2}e^{-i2\pi \varphi }$$

(5) φ=rand[0,1]

(6) $$f(x,\;y,\;t)=F^{-1}(Ff)(x,\;y,\;t)$$

where ξ is the spatiotemporal frequency vector represented in space (x,y) and time $(t),S\left(\cdot \right)$ is the power spectra, β is a set variable of 0, 1.5, 2.5, 3.14, 3.5, or 4.0; F is the Fourier transform; and f(x,y,t) in the value of each pixel in space and time. Increasing values of β decrease the slope of the power spectrum, where a β value of 0 yields a white spectrum (used here for 80 by 60-pixel stimuli), and higher values of β yield more naturalistic spectra with higher spatial and temporal correlations (Simoncelli & Olshausen, 2001). See Extended Figure 1-1 for illustrated examples.

### Electrode implantation

LGN recordings were made with acutely inserted electrodes (n=99 recordings; 77 multichannel recordings, 22 single-channel recordings; three animals) or chronically implanted electrodes (n=12 recordings; one animal). Acute electrodes were either a custom single linear 16-electrode array with 200 μm or 260 μm channel spacing, a linear 16-electrode tetrode configuration array (four groups of four contacts) with 50 μm intra-group spacing and 450 μm inter-group spacing (Plexon U-Probe), or a single traditional tungsten electrode (FHC, Inc.). Chronic electrodes were custom 64-channel microwire bundles designed to splay at depth.

Before acute penetration, the recording chamber was opened and cleaned, the micromanipulator attached (Kopf Instruments Model 650), and used to advance the electrode to the area of interest. Neural responses were recorded by a data acquisition system (Power 1401, Cambridge Electronic Design) and software (Spike 2, Cambridge Electronic Design) that sampled at 40 kHz for all electrodes and channels, to 16-bit resolution. Unless stated otherwise, the data presented in this project are from electrodes with multiple channels (single-channel electrode data from traditional tungsten electrodes are shown only in Figures 4D and 5C).

### Spike sorting

Extracellular recordings can have spikes from multiple nearby neurons recorded on the same channel, and for multi-electrode channels, spikes from the same neuron may appear on multiple channels. To distinguish spikes from different neurons, extracellular signals were automatically sorted through KiloSort 2.5 (Pachitariu et al., 2016). The output was then manually curated with *phy* (Rossant et al., 2016). During manual curation, clusters were cleaned by drawing a boundary in the principal component analysis (PCA) space to remove any abnormal waveforms. The clusters were then assigned a label of *good* or *noise. Good* clusters showed evidence of a refractory period (longer than 1 ms) in the autocorrelogram with a dip to approximately zero, and clear isolation from other clusters in PCA space. Merges were made between *good* clusters if they had similar waveforms (overlapping PCAs), a shared refractory period (a central dip in their crosscorrelogram similar to that from their autocorrelograms), and possibly if there was evidence of drift (one cluster halts spiking when the other starts at an adjacent channel). The lack of an autocorrelogram-like shape in the crosscorrelogram would disqualify a merge. All *good* clusters were identified as single units (SU). The remaining *noise* clusters were not included in additional analysis. See Extended Figure 2-1 for spike sorting examples.

### Extracellular spike waveform analysis

We will represent a single unit’s extracellular signal by its mean extracellular spike waveform, which is then analyzed and compared to the mean waveform of other single units. To obtain the mean waveform of each single unit, u, up to 2,000 spike waveforms, k, were extracted from the raw signal, randomly selected from identified firing times for that neuron and collected as a set $W_{u}^{k}$ (Figure 1C). Each waveform was extracted from a window of 60 samples (1.5 ms) before to 120 samples (3 ms) after the selected spike time; extracted single waveforms were then averaged together to obtain the mean waveform $\overset{-}{W_{u}}$. If a single unit produced less than 2,000 spikes, then all spikes were used (this limitation occurred with 44% of units). For multi-channel electrode recordings, all channels were sampled and averaged, with the single channel with the largest mean spike waveform amplitude selected as the mean waveform for each of those cells. Through the remainder of this report, we will use *spike waveform* to refer to the mean extracellular waveform of a single unit, $\overset{-}{W_{u}}$ from the channel with the largest signature. The recording quality of single units was quantified by a signal-to-noise ratio (SNR; Kelly et al., 2007); the population median of these values was 2.85 (n=303).

### Spike waveform classification

Mean waveforms, $\overset{-}{W_{u}}$, were automatically classified using WaveMAP (Lee et al., 2021), which uses Uniform Manifold Approximation and Projection (UMAP; McInnes et al., 2020) for dimensionality reduction and the Louvain method for cluster identification (Blondel et al., 2008). As in Lee et al. (2021), before WaveMAP classification, the mean waveform of each single unit, $\overset{-}{W_{u}}$, was normalized so that the maximum of its absolute value was 1.

### Receptive Field Analysis

#### Spike-triggered averaging

Each single unit’s chromospatiotemporal (CSTRF, or just RF) was estimated by computing a spike-triggered average (STA; Schwartz et al., 2006), which averages the visual stimulus around each spike time by the following formula:

Eq. 1 $$STA=\frac{1}{N}\sum_{i}s_{i}$$

where $s_{i}$ represents the stimulus in a fixed-size temporal window spanning the time of the *i*^th^ spike, and N is the total number of spikes. The STA was computed using 25 stimulus frames before to 10 frames after each spike (i.e., time lags of −25 to 10 frames, or −156.25 ms to 62.5 ms in steps of 6.25 ms). The entire STA was then whitened by multiplying it with the stimulus covariance matrix (Sharpee, 2013). The resulting chromospatiotemporal receptive field is in four dimensions: time, position in the horizontal dimension, position in the vertical dimension, and color by phosphor (red, R; green, G; blue, B).

#### RF position and latency

The position of each RF was determined by the location of the maximum excursion across the RF. The process of identifying RF position was performed iteratively: a first pass was used to find the absolute maximum; a second pass, to determine the background noise after clipping out a rectangular region 4 degrees on a side centered spatially at the maximum and spanning all time; and a third pass, to enforce statistical selection constraints based on *z*-score. The localization of the peak value was performed in luminance space after converting the CSTRF by taking the vector length of the RGB components for each pixel:

Eq. 2 $${Lum}_{x,y,t}=\sqrt{{R_{x,y,t}}^{2}+{G_{x,y,t}}^{2}+{B_{x,y,t}}^{2}}$$

Eq. 3 $$RF_{location\;}={argmaxLum}_{x,y,t}$$

where R,G, and B are the intensity of the colored phosphors red, green, and blue, respectively, and x,y, and t are locations in the CSTRF matrix in x-space, y-space, and time, respectively. The luminance matrix, Lum, was determined by the magnitude of the colored phosphors. From Lum, the 3-tuple representing the position of its maximum was recorded as the spatiotemporal location of the RF. Throughout this report, RF *position* refers to the x and y spatial components of $RF_{location},RF$ *latency* refers to its temporal component, and $RF_{max}$ to the spatial plane at the latency point. $Lum_{max}$ is the luminance value of $RF_{max}$.

#### RF size and RF spatial window

The RF size and spatial window were measured from the spatial envelope of $Lum_{max}$. First, a 2D Gaussian fit was applied to $Lum_{max}$, and then the length and width of the RF were defined as the full width at 60% maximum (i.e., one standard deviation) along the vertical and horizontal directions, respectively, and corrected for cosine error. The RF size was defined as the average of length and width, and the RF spatial window as the area length times width. All data referring to RF size in this study are from receptive fields from which a clean spatial envelope could be extracted when mapped using high-resolution stimuli.

#### RF temporal window

A temporal window was used to select frames around the peak by fitting a two-term Gaussian model to $Lum_{x,y}$ at the RF position, through time. If the polarity of $RF_{max}$ was *off*, pixels were multiplied by −1 before fitting. Frames where this value at least one standard deviation above the noise level and were of the same polarity as $RF_{max}$ were considered the RF temporal window.

#### *Z*-score

To evaluate the strength of each RF and impose statistical constraints, we computed its *z*-score, its amplitude *a* divided by the noise level η. The amplitude was computed as an un-normalized peak value by averaging the CSTRF pixels within the spatial and temporal windows. This average value, an RGB triplet, was then transformed to luminance space to obtain the RF amplitude a. The noise floor η was determined by the standard deviation of amplitude computed in the same manner with an equivalent number of pixels, but taken randomly from $RF_{noise}$, the acausal frames (frames after the spike, thus positive latencies) of the CSTRF, and repeated without replacement until all pixels in $RF_{noise}$ were used. Normalizing the amplitude by the noise floor yielded the *z*-score for the RF: z=a/η.

#### Cone Weights Calculation

We used the method described in Horwitz & Albright (2005) to obtain the RF color weights (RGB; red, green, and blue phosphor intensities, also known as *gun values*) and then converted those values to cone weights (ℒℳ𝒮; corresponding to sensitivities for long, medium, and short wavelength cones). The color weights were obtained by computing the mean STRF value within the spatial and temporal windows and then converting to cone weights by the following formula:

Eq. 4 $$[LMS]=A^{-T}\;*\;{\left[RGB\right]}^{T}$$

where $A^{-T}$ is the inverse transpose of the three-by-three transformation matrix, A. To obtain the transformation matrix, RGB gun values from the CRT were computed for each color channel in independent trials. Spectral radiance values were measured (PR650 SpectraScan Colorimeter, Photo Research, Inc), and the transformation matrix was obtained from pairwise inner products of the monitor spectral radiance values and 10° cone fundamentals (Stockman & Sharpe, 2000). The ℒℳ𝒮 cone weights were then normalized by dividing each cone weight by the sum of their absolute weights.

##### Cone weight noise

For each of the three cone weights in an RF, a corresponding noise level was measured to determine significance. The noise levels were calculated analogously from the acausal pixels presumed not to contain signal as described for η above, except, instead of transforming the averaged pixels into luminance space, those pixels were transformed into cone space using Equation 4. The noise threshold for each cone weight was then taken as one standard deviation of the corresponding cone space values derived from acausal pixels.

### Response metrics

#### PSTH responsiveness and response latency

The peristimulus time histogram (PSTH) was used to determine responsiveness and response latency for each cell. The PSTH was computed during the time range 600 ms before stimulus onset through the end of stimulus presentation, binned to 1 ms. Responsiveness was determined by comparing bins that were 600–0 ms before to those 200–800 ms after stimulus onset (paired Student’s t-test, *p* < 0.05). When estimating the stimulus response latency, the PSTH was smoothed by applying a running average over a sliding window of length 5 ms, and the response latency was determined by the delay between stimulus onset and when the firing rate crossed 50% between baseline and maximum value during stimulus presentation.

#### Burst index

To describe the firing patterns of LGN cells, we used a burst index which measured how bursty (bunched together in time) or tonic (evenly spread out) a spike train is. Bursts were defined by an inter-spike interval of greater than 100 ms, followed by a string of inter-spike intervals of less than 4 ms. The burst index was then computed as the ratio of spikes occurring in a burst over all spikes in the spike train (Sherman, 2001; Wang et al., 2006).

#### Single Unit Screening

To remain agnostic about cellular responses while maintaining confidence that recorded cells were from within LGN, we used a screening criterion that started with each well-isolated cell for which an RF was found (*n* = 228; RF units) and then included all other well-isolated cells appeared on the same channel (*n* = 75; non-RF units). This screening method resulted in 303 units from 89 experiments spanning three animals.

#### Statistical Analysis

Several statistical analyses and significance comparisons are made throughout this study. All results are shown as the mean with standard deviation (SD), except for Figure 4B, which uses mean with standard error of the mean (SEM). Significance tests involving the mean were done with unpaired Student’s *t*-tests unless stated otherwise. All statistical analyses were performed using MATLAB.

## Results

### Extracellular spike waveforms

A total of 303 single units (SUs) were recorded from 89 electrode penetrations in the LGN of three macaques (M_CH_, *n* = 104; M_ST_, *n* = 51; M_VG_, *n* = 148) and were classified using WaveMAP (Lee et al., 2021), yielding seven distinct spike waveform classifications. Four exhibited typical negative-spiking waveforms (Figure 2 left side): a dominant negative deflection followed by a smaller and slower positive deflection. The differences between these four groups are their width (i.e., trough-to-peak duration) and the magnitude of positive deflection (trough-to-peak ratio). Negative-spiking waveforms are often classified into broad- and narrow-spiking classes, which have been associated with excitatory and inhibitory neurons, respectively (Barthó et al., 2004). Therefore, we defined these four classes as *Narrow 1* (N1, *n* = 39, 13%, shown as dark blue in Figures 2 and 5), *Narrow 2* (N2, *n* = 67, 22%, purple), *Broad 1* (B1, *n* = 49, 16%, orange), and *Broad 2* (B2, *n* = 19, 6%, light blue).

In addition to the typical negative-spiking waveforms, a subset of units exhibited atypical spike waveforms (visualized in the insets under the grouped waveforms within Figure 2). For example, within the four negative-spiking classes, a small population were doublet-spiking waveforms (8%, *n* = 14/174; see right insets of N2 and B2 in Figure 2), where the spike waveform consisted of two distinct negative deflections (examined qualitatively). These doublet-waveforms were predominantly observed in N2 units (67%, *n* = 8/12), with fewer instances in B1 (8%, *n* = 1/12, and B2 units (25%, *n* = 3/12), while no occurrences were noted in N1 units. See Discussion for possible justifications of doublet waveforms along with all other spike waveforms mentioned in this study.

The fifth waveform class, Triphasic (T, *n* = 38, 13%, green), was also negative spiking but had the addition of an initial positive deflection preceding the primary, negative deflection, and subsequent positive deflection. Even though we defined this group as triphasic, not all units had three distinct phases when examined qualitatively (76% qualitatively triphasic, *n* = 29/38; see Figure 2 insets for examples).

The remaining two classes were positive spiking, which we defined as *Positive 1* (P1, n = 47, 16%, black) and *Positive 2* (P2, *n* = 44, 15%, red). We noticed two main differences between these two positive classes. Firstly, P1 waveforms featured a larger negative deflection than those from P2; secondly, P2 as a class had a higher proportion of waveforms with two prominent positive peaks surrounding a negative deflection (i.e., M-shaped; see examples in Figure 2 under the grouped waveforms). The P1 and P2 classes had 9% (*n* = 4/47) and 45% (*n* = 20/44) M-shaped positive spikes, respectively, when qualitatively examined. These positive and triphasic-shaped waveforms, although less commonly reported, have been linked to action potentials propagating through axonal fibers (Meeks et al., 2005; Gold et al., 2006; Lewandowska et al., 2015) and afferent fibers originating from preceding processing areas (Barry, 2015; Sun et al., 2021; Sibille et al., 2022), i.e., from the retina in our case here.

### Receptive fields: RF and non-RF units

All 303 SUs were presented with chromatic noise visual stimuli (see Methods) to estimate their chromospatiotemporal receptive field using spike-triggered averaging. SUs with successful CSTRF, defined by a *z*-score of greater than three (see Extended Figure 3-1A for *z*-score distribution), were categorized as *RF units* (*n* = 228/303, 75%), and nearly all CSTRFs had a lower spatial variance than the gaze location (*n* = 141/142, 99%; Extended Figure 3-1B). The remaining SUs without successful CSTRF mapping are termed *non-RF units* or *N units* (*n* = 75/303, 25%). Both RF and N units were analyzed in this study.

Two stimulus resolutions, coarse and fine, were used during RF mapping (no cell was presented with both). The first two rows in Figure 3 illustrate two example RFs, one from a coarse stimulus (RF 1) and one from a fine stimulus (RF 2). Both examples feature an *on*-center (responding to RGB increments) accompanied by an *off* rebound (responding to RGB decrements). These example cells demonstrate how fine stimuli allowed measurements of RFs with higher spatial resolution. However, we found that this advantage came with the cost of a lower success rate at observing significant RFs than when using coarse stimuli (fine: 70%, n = 158/226; coarse: 91%, n = 70/77), likely due to the higher contrast in the coarse stimuli than the fine stimuli. This is evident in the surround estimated in RF 1 at −31.25 ms compared to the weak indication of a surround in RF 2. Since RF 1 was one of the few RFs that had a clear surround in our measurements, we will concentrate on RF centers.

### M, P, and K classifications of RF units

The 228 mapped RFs were classified into magnocellular (M), parvocellular (P), or koniocellular (K) based on their relative responses to long-, middle- and short-wavelengths (ℒℳ𝒮 cone weights; Eq. 4). Firstly, RFs were classified into koniocellular if the 𝒮-cone weight exhibited the largest magnitude (K, *n* = 21, 9%; see RF 5 in Figure 3 for an example); secondly, parvocellular if the ℒ- and ℳ-cone weights displayed opposing and significantly above noise responses (P, *n* = 69, 30%; RF 3 and 4 in Figure 3 for examples; see Discussion for justification of this definition of parvocellular response); and lastly, all remaining RFs were classified as magnocellular (M, *n* = 138, 61%; RF 1 and 2 for examples), i.e., ℒ- and ℳ-cone weights that were of equal sign. It is important to note that this LGN cell classification is based on functional considerations, which are closely related to the anatomical classifications documented in prior LGN studies (Wiesel & Hubel, 1966; Derrington et al., 1984; White et al., 1998; Reid & Shapley, 2002).

#### M, P, and K cone response characteristics

The normalized ℒℳ𝒮 cone weights of all 228 RFs are visualized in the diamond plot of Figure 4A, a method employed in previous studies comparing LGN neuron cone input (Derrington et al., 1984; Reid & Shapley, 2002; Horwitz et al., 2007). M RFs exhibited an even distribution across the ℒℳ -on and ℒℳ -off planes (Fig 4., top-right and bottom-left lines, respectively), indicating varying contributions from both ℒ- and ℳ-cones. In contrast, P RFs are clustered around the midpoint along the ℒ-on and ℳ-off plane (bottom-right line) but are more evenly spread along the ℒ-off and ℳ-on plane (top-left line). K RFs predominantly displayed 𝒮 -cone *on* responses (*n* = 15/21, indicated by the high proportion of circular purple data points), and half of the K RFs exhibiting an opposing contribution from ℒ- and ℳ-cones (i.e., 𝒮-on and ℒℳ-off, = 10/21) are clustered in the bottom-left quadrant of Figure 4A. The observed 2.5:1 ratio of 𝒮-*on* to 𝒮-*off* RFs aligns with previous studies in marmoset LGN (Martin & Lee, 2014; Pietersen et al., 2014) and macaque retinal ganglion cells (De Monasterio et al., 1975; De Monasterio & Gouras, 1975).

The *temporal weighting function* (TWF), ℒℳ𝒮 response over time for the RF center (Reid & Shapley, 2002), revealed distinct differences among RF classes. M and P RFs exhibited contrasting responses in the ℒ- and ℳ-cone space (M were chromatic non-opponent while P were chromatic opponent), aligning with their role in color vision, with both M and P units showed minimal to no S-cone response (11% and 6% relative to the peak cone response, respectively), also consistent with prior work in macaque LGN (Callaway, 2005) and retinal ganglion cells (Sun et al., 2006). Additionally, M units displayed two clear opposing phases for computing temporal changes, while P units were monophasic for computing ordinary intensity. The TWF of K RFs were monophasic and chromatic opponent, with a moderate contribution from ℒ- and ℳ-cones (29% and 28% relative to peak, respectively).

#### M, P, and K spiking characteristics

Within the RF classes, distinct response metrics differentiate between M, P, and K neurons. First, within 5° of central vision (*n* = 75), the mean RF sizes of M (0.17° ± 0.06°) and P RFs (0.18° ± 0.07°) were significantly smaller than K RFs (0.32° ± 0.06°; *p* < 10^−6^). We also observed that although the rate of activity of M, P, and K units was not significantly different from each other (*p* > 0.27; M, 21.8 ± 15.6 spikes/s; P, 19.5 ± 13.3 spikes/s; K, 17.0 ± 17.1 spikes/s), the spiking activity of M units were more frequent in bursts (burst index of 0.046 ± 0.035) than the more tonic P (0.023 ± 0.023, *p* < 10^−4^) and K units (0.021 ± 0.031, *p* = 0.012). Not only did M units spend more time in bursts, but they also had shorter mean unsigned response latency (31.8 ± 7.8 ms) than P (42.1 ± 11.8 ms, *p* < 10^−8^) and K units (47.5 ± 5.2 ms, *p* < 10^−10^). These findings are supported by previous LGN studies regarding RF size and response latency (Maunsell et al., 1999; Pietersen et al., 2014; Eiber et al., 2018) but present novel insights regarding burst spikes across RF classes (Ruiz et al., 2006; Pietersen et al., 2017).

We also found two unexpected differences between cells classified as M and P by RFs. Due to their larger cell bodies, we expected M units to have had the largest recorded waveform amplitude out of the three classes. This was not the case with the M units in the current study (*p* > 0.37) (Figure 4C fifth row), likely because we did not optimize electrode position for each cell, and thus units were recorded at varying distances from the electrode tips (as amplitude is inversely proportional to source-to-site distance: Holt & Koch, 1999; Gold et al., 2006). Second, the mean estimated depths for M and K units were significantly more superficial than P units (*p* < 0.02) (Figure 4C sixth row), contrary to expectations based on LGN anatomy. It is important to note that the estimated depth is not a complete and accurate measurement of somatic depth (see Discussion for further details).

### Non-receptive field (N) units

A substantial portion (25%, *n* = 75/303) of our recorded population, termed N units, did not exhibit measurable receptive fields despite being recorded alongside units with identifiable RFs. These N units displayed significantly lower spiking activity than RF units (N, 7.3 ± 10.2 spikes/s; RF, 20.6 ± 15.0 spikes/s; *p* = 10^−9^; right side of Fig. 4D), suggesting that that the visual stimulus did not activate these units, however more than half of N units had a significantly elevated firing rate during stimulus onset versus immediately prior (65% of N units; paired t-test; see Methods; 82% for RF units). The lower spiking activity of N units may be due in part to insufficient data for a significant receptive field to be estimated, as RF quality as assessed by *z*-score was moderately correlated with number of spikes in RF cells (*r* = 0.58).

Additionally, N units exhibited more sustained spiking activity than RF units (burst index of; N, 0.015 ± 0.013; RF, 0.036 ± 0.033; *p* = 10^−6^), which was moderately correlated with RF *z*-score (r = 0.45), and were estimated at a depth more superficial than RF units (N, 50.9 ± 2.4 mm; RF, 51.6 ± 2.7 mm; *p* = 0.049), suggesting that anatomical location may be relevant to the recording of non-RF units. For the remaining response metrics, N units had no significant differences in response latency compared to RF units (N, 47.2 ± 20.4 ms; RF, 50.7 ± 25.9 ms; *p* = 0.53; estimated from PSTHs: see Methods), and no significant differences in waveform amplitude (*p* = 0.80), suggesting that the timing and quality of the spikes were irrelevant to the recording of non-RF units.

We observed two experimental factors that may have influenced the N unit population. By including the single-channel units that were initially excluded from the dataset (*n* = 48) because of potential technologically-driven sampling biases (Talebi & Baker, 2016), N units were more commonly recorded with single-channel electrodes than multi-channel electrodes (44%, *n* = 21/48, versus 25%, *n* = 76/303; Figure 4E left panel). This discrepancy is likely due to a difference in electrode technology, as the single-channel electrodes used were sharper and had higher impedance than the multi-channel electrodes, reinforcing our initial concern to exclude single-channel units (see Discussion for further details). Moreover, the proportion of N units recorded with coarse stimuli was much lower (9%, *n* = 7/77) than with fine stimuli experiments (30%, *n* = 68/226; Figure 4D right panel), likely due to the stimulus’ larger pixel size and higher contrast: driving neurons more strongly and perhaps driving a larger proportion of the population than the fine stimuli.

Overall, our findings reveal a population of non-RF units with distinct response characteristics and dependencies on electrode type and stimulus type, shedding light on previously unreported aspects of neural activity in the LGN.

### Correlations

Correlating spike waveform classifications (N1, N2, B1, B2, T, P1, and P2), RF classifications (M, P, K, and N), and various response characteristics (e.g., spike-rate, response latency, RF size) revealed intriguing patterns and insights into LGN neural processing. When examining spike waveform classifications alongside RF classifications, several notable observations emerged: (*i*) the narrow negative-spiking classes (N1 and N2) were predominantly associated with P RFs (36–46%) with approximately a 2-to-1 ratio to M RFs for N1 units and a 1-to-1 ratio for N2 units; (*ii*) the broad negative-spiking classes (B1 and B2) had the largest proportion of N units (37–47%); and (*iii*) the positive-spiking classes (P1 and P2) were mostly linked to M RFs (66–68%) with a 5-to-1 ratio to P RFs and the least proportion of K RFs (0–4%). This heterogeneity indicates there may be functional differences between waveform classes in the LGN but may also reflect sampling biases inherent in the recording process (Towe & Harding, 1970). For example, the higher proportion of M RFs from the positive-spiking units may indicate the presence of more axonal projections from the deeper M layers (passing upward through the P layers as they flow toward the optic tract) than the P and K projections in our recordings.

Further comparative analysis of response characteristics reinforced these findings. (Figure 5B). The two positive-spiking classes (P1 and P2) responded to the visual stimuli with a significantly shorter response latency than the four negative-spiking classes (N1, N2, B1 and B2) (*p* < 0.004); the RF size of narrow negative-spiking classes (N1 and N2) were significantly smaller than all other classes (*p* < 0.03); and positive and triphasic (P1, P2 and T) classes had RFs at significantly larger eccentricities the negative-spiking classes (*p* < 0.003). These observations are particularly significant because the shorter response latencies of the non-classical positive-spiking classes support the notion that these waveforms originate from axons in the retinothalamic projection, and, if the recorded positive waveforms were axonal, then it accounts for their higher eccentricities as eccentricity is positively correlated with axon diameter (Walsh et al., 2000). Therefore, our observations show that the non-classical positive-spiking waveforms recorded in the LGN have distinct functional characteristics to the classical negative-spiking classes, similar to previous studies (cat V1: Sun et al., 2021).

Moreover, we also noted two additional metrics that may be of interest (Figure 5B last two rows): overall spike size (amplitude; trough-to-peak magnitude) and recording position (estimated depth). N1 waveforms exhibited the largest mean amplitude of all other classes (*p* < 0.001), signifying that N1 may represent recordings from large neurons or recordings of cells that were near the electrode (Holt & Koch, 1999; Gold et al., 2006). In terms of the anatomical recording positions, three out of the four classical negative-spiking classes (N2, B1, and B2) were recorded significantly deeper than the positive-spiking and triphasic classes (T, P1, and P2) (*p* < 0.035), indicating differences in the anatomical origin of these waveforms. The more superficial position of positive and triphasic units suggests they were from thalamocortical or corticothalamic axonal fibers traveling to or from the LGN, with thalamocortical projections being more frequent due to the significantly faster response latencies of the positive-spiking groups.

Methodological factors, such as electrode type and stimulus type, also influenced the distribution of waveform types (Figure 5C). Single-channel electrodes predominantly recorded negative-spiking units (96%, *n* = 46/48), while multi-channel electrodes captured a more diverse range of waveform types, perhaps due to the dissimilar electrode properties or selection bias for strong signals when using single-channel electrodes as opposed to multi-channel electrodes (Talebi & Baker, 2016). Additionally, coarse stimuli resulted in a higher proportion of positive-spiking units compared to fine stimuli (46% versus 24%), highlighting a dependency between stimulus type and recorded waveform types.

## Discussion

### Extracellular spike waveforms

Our investigation into extracellular spike waveforms within the LGN reveals a diverse array of classes, each with distinct characteristics. The negative-dominant waveform classes (N1, N2, B1, B2) exhibit variations in spike duration, attributed by the opening and closing of sodium and potassium channels, and occur when the recording contact is near the neuron’s soma with minimal influence from other structures (Holt & Koch, 1999; Gold et al., 2006). The variations is spike duration has been linked to neuronal type, with broad-spiking waveform associated with excitatory neurons and narrow-spiking associated with inhibitory neurons (Henze et al., 2000; Barthó et al., 2004; Sukman & Stark, 2022). We have not made this assumption for our dataset because excitatory cells have exhibited narrow-spiking extracellular waveforms (Vigneswaran et al., 2011), and inhibitory interneurons in mouse cortex have exhibited broad-spiking waveforms, intracellularly (Gentet et al., 2010).

The upward deflection in triphasic- and positive-spiking waveforms has been described as the mixed-ion capacitive current from surrounding sources for recordings distal to the soma as the spike propagates through an isolated unmyelinated axon (Clark & Plonsey, 1968; Raastad & Shepherd, 2003; Heinricher, 2004; Gold et al., 2006; Lewandowska et al., 2015). Furthermore, previous studies have shown that only triphasic- and positive-spiking waveforms were recorded from silenced brain regions (Barry, 2015; Sun et al., 2021; Sibille et al., 2022), demonstrating that these waveforms are of afferent fibers originating from previous processing areas. Our findings are consistent with these reports, as the positive waveforms (P1, P2), and to a lesser extent the triphasic waveforms (T), had shorter response latencies than the negative-spiking classes, suggesting they reflect axonal fibers of passage of retinal ganglion cells.

Although the waveform clustering algorithm used here provided well-defined waveform groupings, there were multiple variations within each waveform class. Within the negative classes, a small population were doublet-spiking waveforms that may reflect retinal spikes impinging on LGN neurons (S-potentials: Sincich et al., 2007), or intermediate LGN neurons like those found in ferret LGN (Murphy et al., 2020). Within the positive classes, we noticed several M-shaped waveforms (double positive peaks), which could also be from S-potentials or from distal neuronal activity such as from dendrites (Holt & Koch, 1999; Gold et al., 2006). Due to their rarity, we did not investigate if these unusual waveforms functionally differed from others. Such subpopulations are rarely reported in the literature and indicate potential complexities in extracellular action potentials that warrant further investigation.

### RF classification

Our study revises the traditional RF classification method (Wiesel & Hubel, 1966; Reid & Shapley, 2002), to account for experimental uncertainties: i.e., for a RF to be considered P, both opposing ℒ- and ℳ-cones had to be significantly above noise. This refinement is important because, when classifying our RFs using the traditional method (sign only), we found several RFs that qualitatively appeared to be magnocellular, but would have been labeled parvocellular by classical criteria due to ℒ- or ℳ-cone responses within the noise floor, resulting in an inaccurate classification (see Extended Figure 3-2 for examples). Our new classification is more robust in the face of experimental uncertainty while preserving the functional definitions.

We reported above (Figure 4C) that magnocellular units (sorted spike trains leading to M RFs) were unexpectedly found more superficial than parvocellular units (those leading to P RFs), which is not what is traditionally held. Bearing in mind that we likely recorded signals of both somatic and non-somatic origin, we found magnocellular units consisting of both negative-spiking and positive-spiking units, and that these sub-classes differed in depth. The positive-spiking magnocellular units were recorded at estimated depths significantly more superficial to those with negative waveforms, suggesting that magnocellular units with positive waveforms were axonal activity traversing through the LGN. In fact, when considering only the negative-waveform magnocellular units, there were no significant differences in depth to those that lead to P or K RFs.

### Correlations

Distinct correlations between extracellular spike shape and RF characteristics were observed. First, the narrow-spiking classes (N1 and N2) had a 1.5-to-1 ratio of P-to-M RFs, as often seen in LGN extracellular recordings (Pietersen et al., 2014). Even though this ratio is much lower than the 8-to-1 ratio of the parvocellular-to-magnocellular population in the LGN (Prasad & Galetta, 2011), the extracellular ratio may be due to a sampling bias of extracellular recordings towards the larger magnocellular neurons (“the larger the fish, the higher the probability of a hit” — Towe & Harding, 1970).

Second, positive-spiking waveforms (P1 and P2) had a 1-to-5 ratio of P-to-M RFs. If we assume positive spikes are afferent axons from the retina, and the LGN has a 1-to-1 ratio to retinal ganglion cell input (Spear et al., 1996), then why are the ratios of RF classes in the positive and negative classes unequal? One might expect that negative and positive spikes are functionally different, but we think that sampling biases were involved when recording from axonal activity. In particular, factors including the generally larger diameter axons from parasol/magnocellular neurons compared to those of midget/parvocellular and koniocellular cells (Walsh et al., 2000), the larger magnocellular-to-parvocellular ratio as eccentricity increases (Livingstone & Hubel, 1988), differential degrees of axon myelination (Holt & Koch, 1999), and the prevalence of magnocellular LGN axons in upper layers as they course through the interior of the area toward the primary visual cortex, could have biased our recording of axonal (positive spike) signals toward the magnocellular population.

Third, N RFs (those without an estimated RF) were found with higher proportion in the broad-spiking waveform classes (B1 and B2) compared to all other waveform classes. We observed that N RFs had many response characteristics that differed from units with an estimated RF (discussed further below).

### Non-RF units

The presence of N units raises intriguing questions regarding their identity and functional significance. We found that almost two-thirds of N units responded reliably to the stimulus albeit at a reduced spike and burst rate than RF units. The shortage in spiking activity could be from the electrode channels losing contact with the source, which may explain the high proportion of N units in the broad-spiking population, as contact distance is proportional to spike waveform width (Gold et al., 2006). The shortage of burst activity for N units may be associated with a lack of stimulus contrast processing (Sanchez et al., 2023), higher label information processing (Butts et al., 2010), or a lack of retinothalamic transmission of visual information (Alitto et al., 2019). Together, this evidence from the literature can suggest that some of the N units may have been corticothalamic projections (evident in the more superficial recorded depth), such as those that influence response gain (Murphy et al., 2021).

For the remaining N units that did not reliably respond to the stimulus (43%), what do these units do if they are not being visually stimulated? As here, Vries et al. (2020) found 23% of mouse visual cortex neurons (~60,000 total) did not reliably respond to visual stimuli, with the proportion of non-responsive units increasing in higher visual areas. They speculated that these units might be involved in specific natural features from hierarchical processing, modulated by multimodal senses (Stringer et al., 2019), or non-visual computation, such as motion, identified in the dLGN of mice (Orlowska-Feuer et al., 2022), which may be true for our N units. It is also possible that these N units may explain the retinal ganglion cell classes projecting to the LGN that are not associated with classical thalamic responses (~20%: Dacey, 2004). While it may be simply that there was insufficient activity for a significant RF to be recovered here, gaining insight into non-responsive units in future studies is integral to a complete understanding of visual processing (Olshausen & Field, 2005).

### Evidence for recording bias

Differences in populations between our multi-channel and single-channel recordings is likely due to technologically-driven sampling bias. Talebi & Baker (2016) postulated that manually controlled single-channel electrodes in combination with a search stimulus to identify responsive neurons, largely avoided in our multi-channel recordings, creates a selection bias toward user-preferred neurons. Similarly, previous studies utilizing single-channel electrodes may have overlooked atypical positive-spiking units when searching for neurons, especially when using a negative trigger threshold when searching for neurons or during off-line spike-sorting.

It is important to note that the current study should be viewed as a partial survey of the LGN, rather than an exhaustive one. We searched while listening for spikes along with looking for them, often using a negative trigger threshold. This strategy may have created a selection bias on the channel being monitored despite our attempts to mitigate those effects, and the true proportion of positive-spiking waveforms may be higher than what we have reported. For example, Paulk et al. (2022) recorded more positive-spiking than negative-spiking units in human cortex using dense multi-channel electrodes. Several other experimental biases are difficult to avoid in extracellular recordings, such as preference for large extracellular action potentials, units with high firing rates, or units that are visually responsive to the stimulus (Olshausen & Field, 2005). Thus, future studies need to consider the possible biases when recording extracellularly to overcome the difficulties involved in obtaining a completely objective study of a neuronal population.

### Conclusion and implications

In conclusion, our study sheds light on the complexity of LGN neuronal populations, challenging traditional classifications and highlighting the presence of previously undocumented LGN units such as non-RF units. These findings expand our understanding of LGN function and offer significant implications for future research, particularly elucidating the intricacies of extracellular signals, addressing recording biases, and providing a deeper understanding of receptive field data in the awake macaque LGN, for which there is limited information at the high spatiotemporal resolution presented in this study. Furthermore, our work opens avenues for interdisciplinary exploration, with potential applications in broader neuroscience research (e.g., comparing function to principal cell types through transcriptomics: Bakken et al., 2021). Overall, our study contributes valuable insights into LGN function and underscores the need for continued investigation into its neural dynamics.

## Extended Data

**Figure 1–1.**
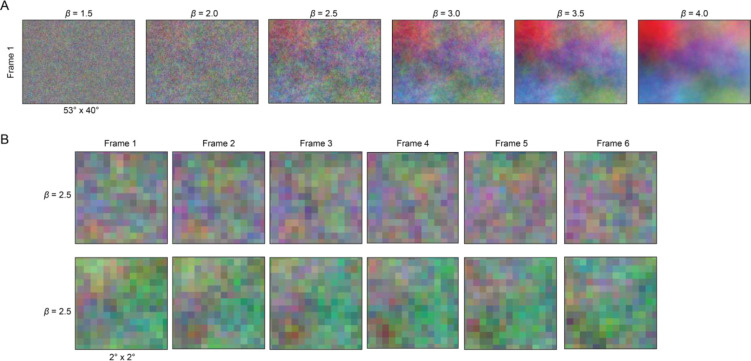
Visualizing the range of spatiotemporal correlations created by increasing values of β. (A) Example single frames from stimulus sets for β = 1.5, 2.0, 2.5, 3.0, 3.5, and 4.0. As β increases, so does the spatiotemporal correlation. (B) Example frames from two independent stimulus sets with β = 2.5. Each row depicts the central 2° for six consecutive frames from a given set.

**Figure 2–1.**
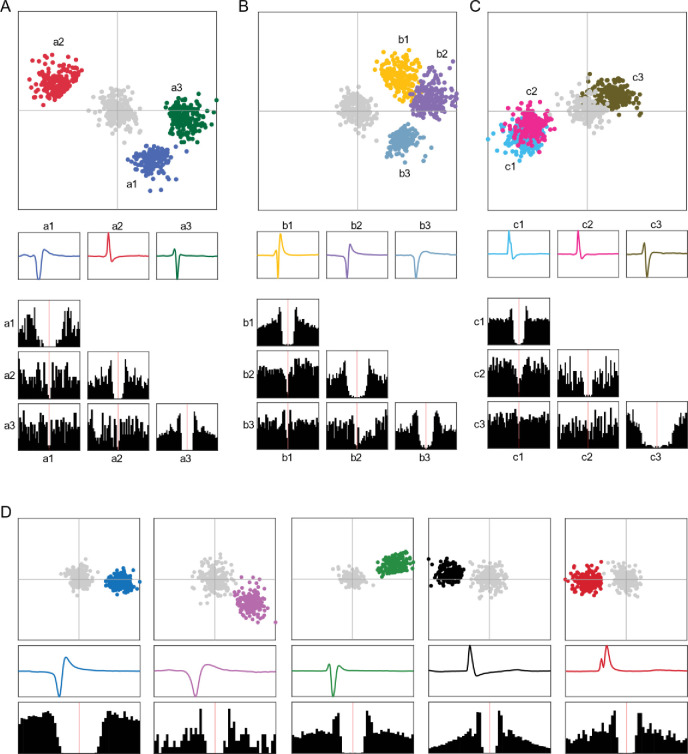
Spike sorting examples. **(A-C)** Three example single-channel recordings, each with multiple isolated units. The top row illustrates the PCA feature space of the spike waveforms (limited in this figure to 200 spikes). The grey cluster is noise. The second row is the mean waveform of each single unit sorted on that recording channel. The third row is the auto- and cross-correlograms between the same example units. **(D)** Five example single units from additional recordings with only one single sorted unit, with the same information as in A-C.

**Figure 2–2.**
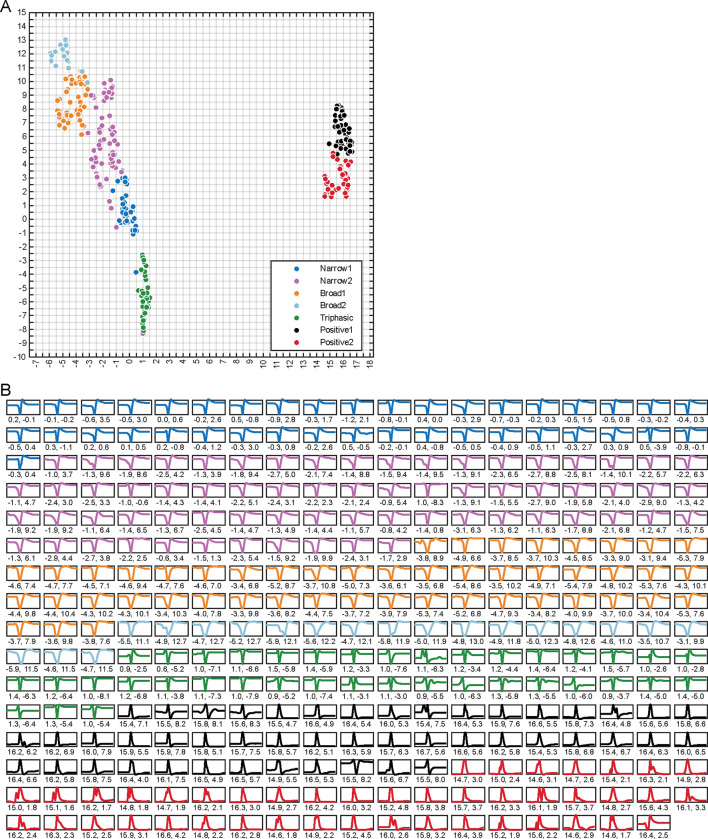
All extracellular spike waveforms plotted in **(A)** UMAP space, and **(B)** mean spike waveform traces for each single unit with their corresponding UMAP coordinates. The seven colors refer to the seven waveform classifications from WaveMAP.

**Figure 3–1.**
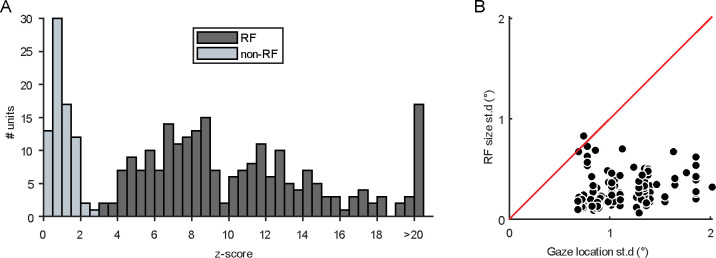
Receptive field statistics. **(A)** Distribution of z-scores across all single units. The histogram is binned in size of 0.5 z-score. The black and grey bins refer to RF and non-RF units, respectively. **(B)** Standard deviation of RF size (°) plotted against standard deviation of gaze position (°). The identity line is plotted in red. Nearly all CSTRFs had a lower spatial variance than the gaze location.

**Figure 3–2.**
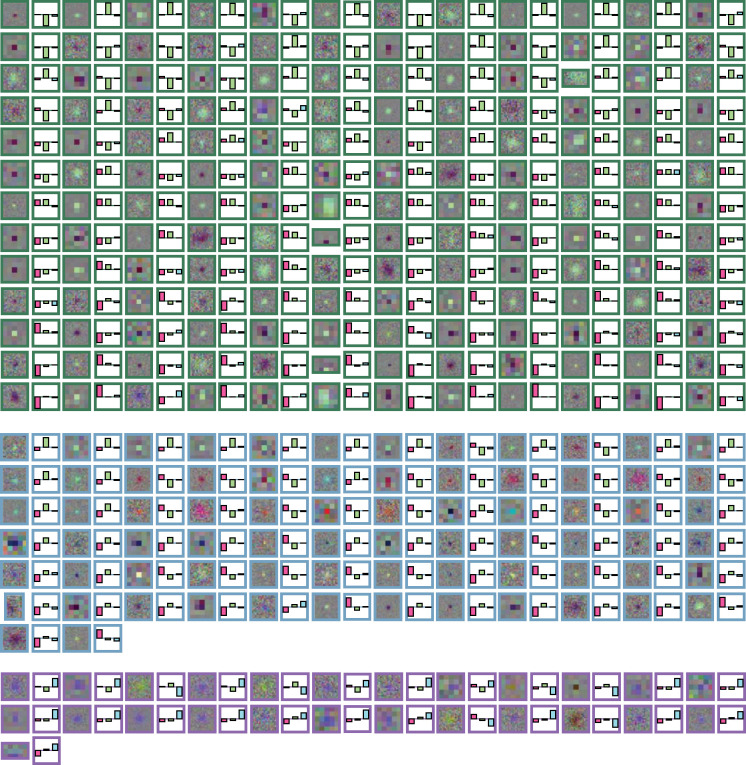
$RF_{max}$ and corresponding cone weight from all RF units. RFs are visualized in RGB, and the three cone weights correspond to L, M, and S. The three groups of RFs are M RFs (green border), P RFs (blue border), and K RFs (purple border). All RF plots span 2° in visual space.

## Supplementary Material

Supplement 1

## Figures and Tables

**Figure 1.**
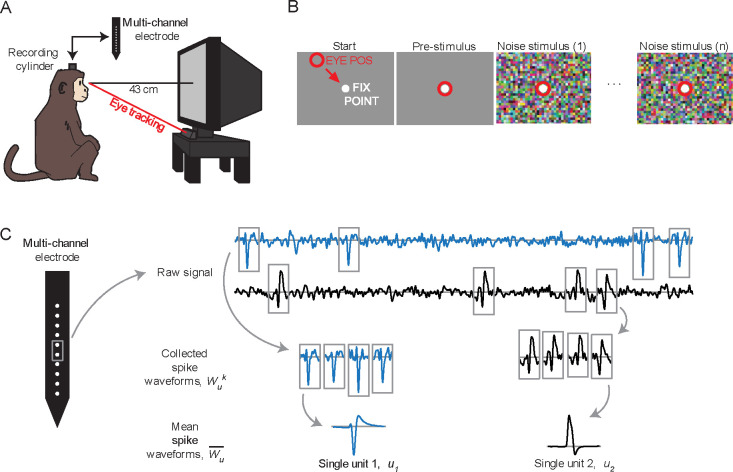
Experimental design. **(A)** Animals were placed in front of a CRT monitor, with neutral gaze position near the center of the monitor. Gaze location was monitored through an infrared eye tracker. Multi-channel electrodes were inserted through surgically-implanted titanium recording cylinders which maintained chronic access to the dura. **(B)** The trial-based mapping task. The animal was required to fixate on a small spot in the center of the screen for 0.4 sec, before the mapping stimulus commenced, and to maintain fixation on the spot for the duration of the mapping stimulus. Mapping stimuli were presented for 2.5 to 5.0 seconds. **(C)** Schematic of extracellular spike waveform extraction. For each single unit (u), spike waveforms were extracted from the raw signal $\left(W_{u}^{k}\right)$ in 4.5 ms windows and then averaged to obtain the mean waveform $\left(\overset{-}{W_{u}}\right)$.

**Figure 2.**
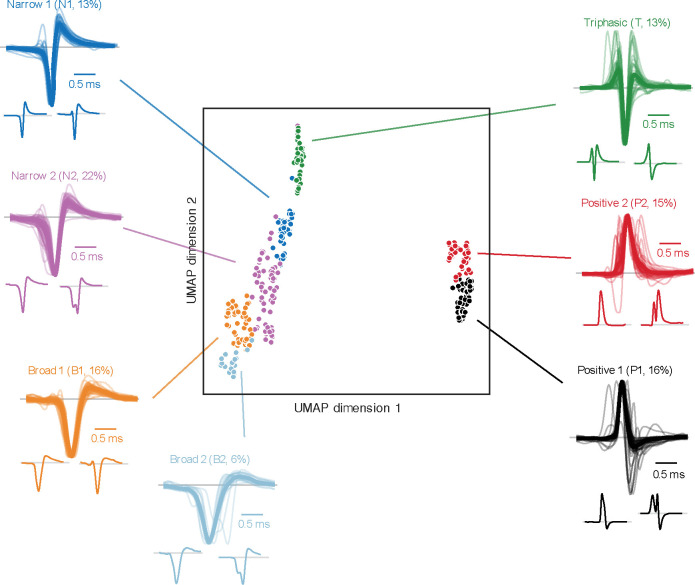
Extracellular mean spike waveforms are classified using WaveMAP (Lee et al., 2021). In the middle, scatter plot visualization of each single unit’s mean spike waveform in UMAP space, where the horizontal and vertical axes represent the dimension-reduced UMAP coefficients. The data points are colored by the seven waveform classifications from WaveMAP: *Narrow 1* (N1, blue); *Narrow 2* (N2, purple); *Broad 1* (B1, orange); *Broad 2* (B2, light blue); *Triphasic* (T, green); *Positive 1* (P1, black); and *Positive 2* (P2, red). Surrounding the scatter plot are the mean spike waveform traces for each single unit (light lines) with the group mean waveform overlayed on top (dark and bold lines), for each class of waveforms. Negative-spiking groups are normalized to the negative peak, and positive-spiking classes (P1 and P2) are normalized to the positive peak. The smaller individual waveforms illustrated below the grouped waveforms are two examples to show the variation within each class. Gray lines within the illustrated waveforms represent baseline voltage. All waveforms were plotted within a 2.5 ms window. See Extended Figure 2-2 for illustrations of all extracellular spike waveforms.

**Figure 3.**
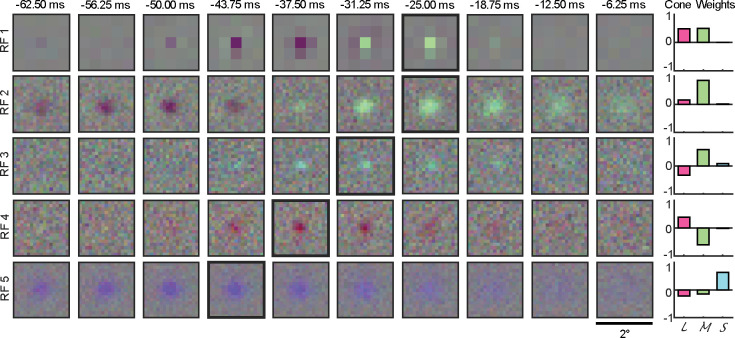
Five example chromospatiotemporal receptive fields (RFs), one per row, spanning 12 stimulus frames, one per 6.25 ms. RF 1 was measured using coarse-resolution stimuli, and RF 2–5 were measured using fine-resolution stimuli. All plots span 2.0° of the visual field. Bolded black outlines indicate the frame in an RF with the largest magnitude $\left(RF_{max}\right)$. On the right are the cone weights for each example, indicated as long- (ℒ, magenta), medium- (ℳ, green), and short-cone wavelengths (𝒮, blue) on the x-axis and the normalized response on the y-axis. See Extended Figure 3-2 for the $RF_{max}$ and cone weights for all RF units.

**Figure 4.**
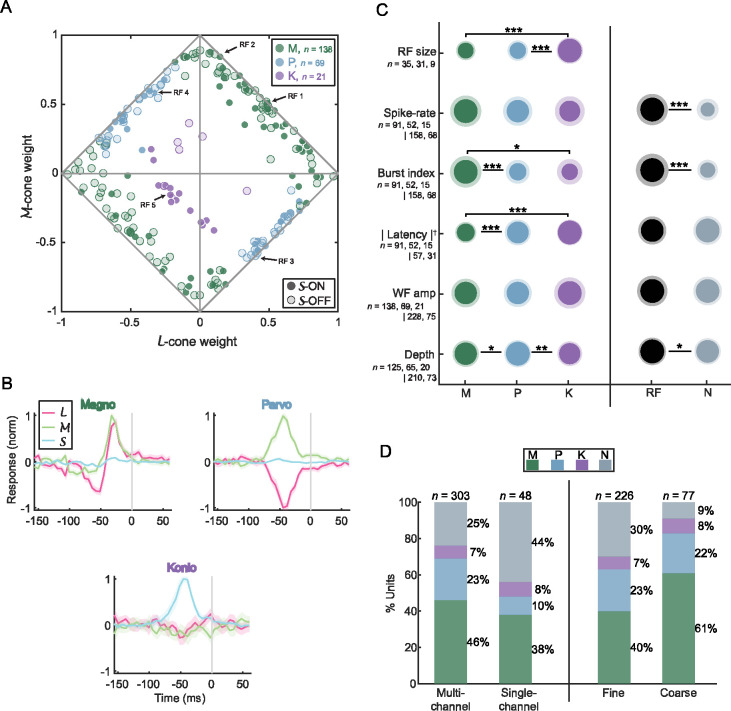
Receptive field classification and analysis (*n* = 228). **(A)** Normalized cone weights (|L| + |M| + |S| = 1) for all RFs plotted on the ℒℳ plane. The horizontal and vertical axes represent ℒ- and ℳ-cone weights, respectively. The magnitude of the S-cone weight is represented as the distance from the diamond lines. The circular and diamond-shaped data points with positive and negative 𝒮-cone weights, respectively. The data points colored in green, light-blue, and purple represent magnocellular (M), parvocellular (P), and koniocellular (K) RFs, respectively (also in subplots C and D). **(B)** Mean temporal weighting functions (TWFs), describing the average cone response over time for M (top-left), P (top-right), and K (bottom) RFs. The grey line indicates spike onset. For M and K RFs, weighting functions were multiplied by −1 for *off* RFs, and for P RFs, responses were flipped if the maximum ℒ-cone response was negative. **(C)** Intensity visualization summarizing the mean value of a response metric for each RF class separated into M, P, and K on the left and RF and non-RF (N) units on the right. The area of the inner and outer circles represents the mean and standard deviation, respectively. The notations *, ** and *** represent *p* < 0.05, *p* < 0.01 and *p* < 0.001 (unpaired t-test), respectively. Note that response latency (indicated by the dagger symbol, †) was calculated differently for the left (from the $RF_{max}$) and right datasets (from the PSTH). Since differences in stimulus statistics have been known to affect spiking metrics (Almasi et al., 2022; Sanchez et al., 2023), the RF size, spike-rate, burst index, and response latency mentioned in this and following sections were from units stimulated with fine-stimulus only. |Latency| refers to the absolute response latency. **(D)** Percentage of units recorded from multi-channel electrodes versus single-channel electrodes (left) and units recorded with the fine stimuli versus coarse stimuli (right) for each RF class.

**Figure 5.**
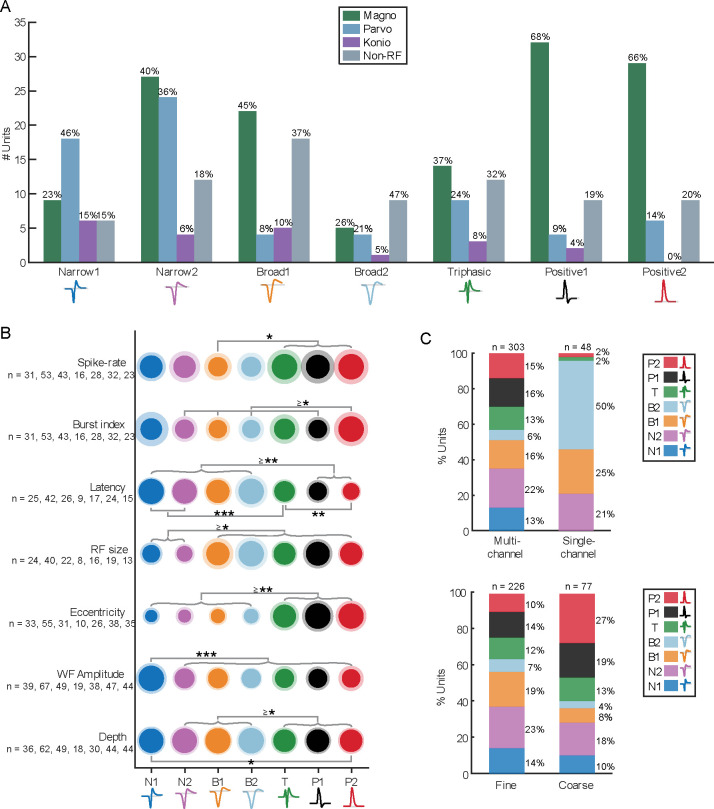
Correlations and analyses of spike waveform classes against RF classes and response characteristics. **(A)** Bar plot of waveform class population to their RF type (M, P, K, N) as number of units (*n* = 303). The percentage above each bar indicates the proportion of each RF type within that waveform class. **(B)** Intensity visualization summarizing the mean value of a response metric for each waveform class, like in Figure 4C. The area of the inner and outer circles represents the mean and standard deviation, respectively. The notations *, ** and *** represent *p* < 0.05, *p* < 0.01 and *p* < 0.001 (unpaired *t*-test), respectively. RF population for each dataset is stated below each tick label along the vertical axis. Spike-rate, burst index, latency, and RF size were estimated from units presented with fine-stimuli. **(C)** The percentage of units recorded from multi-channel versus single-channel electrodes for each spike waveform class. The percentages on the right of each bar denote the proportion of each waveform class, and the number above denotes the total number of units within that bar.

## Significance statement

This study uncovers evidence for an intricate diversity of neuronal responses within the lateral geniculate nucleus (LGN), challenging conventional classifications and revealing previously overlooked populations. By characterizing extracellular spike waveforms and revising receptive field classifications, we provide novel insights into LGN function. Our findings have significant implications for understanding early visual processing mechanisms and interpreting extracellular signals in neural circuits. Furthermore, we identify non-receptive field units, prompting exploration into their functional roles and broader implications for visual and non-visual computations. This study not only advances our understanding of LGN organization but also highlights the importance of considering recording biases in electrophysiological studies. Overall, our work opens new avenues for interdisciplinary research and contributes to advancing our knowledge of neural dynamics in the visual system.
