# Supplementary material for: More than expected: extracellular waveforms and functional responses in monkey LGN
Source: bioRxiv. 2024 May 16:2023.11.22.568065. Preprint. [Version 2] doi: 10.1101/2023.11.22.568065 (PMC11118448; doi:10.1101/2023.11.22.568065)
Supplement: Supplement 1 [file NIHPP2023.11.22.568065v2-supplement-1.pdf]

bioRxiv preprint doi: https://doi.org/10.1101/2023.11.22.568065; this version posted May 16, 2024. The copyright holder for this preprint (which was not certified by peer review) is the author/funder, who has granted bioRxiv a license to display the preprint in perpetuity. It is made available under aCC-BY 4.0 International license.

SUPPLEMENTARY MATERIAL

Appendix S1: Glossary

SU, single unit: isolated extracellular recording from an individual neuron.

CSTRF, chromospatiotemporal receptive field: the receptive field of an LGN cell measured across color and visual spaces, and through time.

RF, receptive field: the response of the optimal stimulus for a given cell, used interchangeably with the CSTRF.

RF unit: a single unit with a significant receptive field.

STA, spike triggered average: the averaged stimulus image conditioned on spike time, here used to calculate the CSTRF.

R, G, B; red, green, blue: the three phosphor colors used in computer monitors and, here, to generate visual stimuli.

Lum, luminance matrix: determined by the magnitude of the colored phosphors.

$RF_{location}$: the location of CSTRF with the maximum, from Lum, in x-space, y-space, and time.

RF position: the x and y spatial components of $RF_{location}$.

RF latency: the temporal component of $RF_{location}$.

RF_{max}: the spatial plane at RF latency.

bioRxiv preprint doi: https://doi.org/10.1101/2023.11.22.568065; this version posted May 16, 2024. The copyright holder for this preprint (which was not certified by peer review) is the author/funder, who has granted bioRxiv a license to display the preprint in perpetuity. It is made available under aCC-BY 4.0 International license.

Lum_{max}: the luminance matrix of RF_{max}.

RF_{noise}: the acausal frames of the CSTRF.

η: noise in a signal; used to select RFs with significant amplitudes.

$\mathcal{L}$, $\mathcal{M}$, $\mathcal{S}$; long, medium, short: retinal photoreceptor cones classified by wavelength of peak sensitivity.

M, magnocellular: RFs with contribution from $\mathcal{L}$- and $\mathcal{M}$-cone weights that were non-opposing.

P, parvocellular: RFs with contribution from $\mathcal{L}$- and $\mathcal{M}$-cone weights that were opposing and significantly above noise.

K, koniocellular: RFs with the largest contribution from $\mathcal{S}$-cone.

N, non-RF unit: cells that do not have significant receptive fields.

TWF, temporal weighting function: the LMS response of a given pixel, or average pixel, over time.

Narrow 1, 2: narrow, negative-going spike waveform classifications (see Fig. 2).

Broad 1, 2: broad, negative-going spike waveform classifications (see Fig. 2).

Triphasic: negative-going spike waveform classification but typically with an addition of an initial positive deflection (see Fig. 2).

Positive 1, 2: positive-going spike waveform classifications (see Fig. 2).

Coarse stimulus: 80 x 60 colored noise stimulus.

Fine stimulus: 400 x 300 colored noise stimulus.
